# Early Hearing-Impairment Results in Crossmodal Reorganization of Ferret Core Auditory Cortex

**DOI:** 10.1155/2012/601591

**Published:** 2012-07-19

**Authors:** M. Alex Meredith, Brian L. Allman

**Affiliations:** ^1^Department of Anatomy and Neurobiology, Virginia Commonwealth University School of Medicine, Richmond, VA 23298, USA; ^2^Center for Hearing and Deafness, State University of New York at Buffalo, Buffalo, NY 14202, USA

## Abstract

Numerous investigations of cortical crossmodal plasticity, most often in congenital or early-deaf subjects, have indicated that secondary auditory cortical areas reorganize to exhibit visual responsiveness while the core auditory regions are largely spared. However, a recent study of adult-deafened ferrets demonstrated that core auditory cortex was reorganized by the somatosensory modality. Because adult animals have matured beyond their critical period of sensory development and plasticity, it was not known if adult-deafening and early-deafening would generate the same crossmodal results. The present study used young, ototoxically-lesioned ferrets (*n* = 3) that, after maturation (avg. = 173 days old), showed significant hearing deficits (avg. threshold = 72 dB SPL). Recordings from single-units (*n* = 132) in core auditory cortex showed that 72% were activated by somatosensory stimulation (compared to 1% in hearing controls). In addition, tracer injection into early hearing-impaired core auditory cortex labeled essentially the same auditory cortical and thalamic projection sources as seen for injections in the hearing controls, indicating that the functional reorganization was not the result of new or latent projections to the cortex. These data, along with similar observations from adult-deafened and adult hearing-impaired animals, support the recently proposed brainstem theory for crossmodal plasticity induced by hearing loss.

## 1. Introduction

Neural plasticity affords the brain the remarkable capacity for adapting to features of its sensory environment. This same mechanism, however, also renders the brain vulnerable to altered or deprived developmental experiences. Under these conditions, the neural representation of a damaged sensory system can be replaced by inputs from the intact sensory modalities, and this substitution of one sensory modality with another is referred to as crossmodal plasticity. To date, most examples of crossmodal plasticity have been observed in subjects that experienced sensory deprivation/loss either congenitally, or early in life [1–3]. For example, following early-deafness, visual crossmodal effects have been documented within secondary or auditory association areas [2, 4–8]. In addition, crossmodal plasticity has been shown to convey supranormal performance in the remaining modalities, such as in tasks of visual spatial localization [8–10] or visual motion detection [10] in early-deaf subjects. Recently it has been shown that the neural bases for these perceptual enhancements in the early-deaf were not distributed homogeneously across the “vacated” auditory cortex, but were dependent on specific subregions of visually reorganized auditory cortex [10, 11]. Because each affected neural area houses the circuitry for a specific behavioral program (localization, movement detection) that is the same in deaf or hearing subjects, and enhanced performances are based on stimulus features that are common to both the auditory and visual modalities (e.g., stimulus location or movement velocity), these observations suggest a supramodal basis for enhanced crossmodal performance, an effect now regarded as Lomber's Law [10]. These same experiments also indicated that some areas of vacated auditory cortex, in particular A1, were not involved in any of the many visual crossmodal tasks examined, an effect that was consistent with numerous studies of early-deaf subjects [2, 4–7, 12]. Thus, early-deafness can lead to supranormal crossmodal performance on specific tasks mediated by particular subregions of auditory cortex, while other regions seem to be unaffected by crossmodal plasticity.

 In contrast to the considerable attention that early sensory loss has received, very few studies have examined the crossmodal effects of late, or adult, sensory loss. Instead, the neural bases for deafness-induced adult crossmodal plasticity were virtually unexplored until recently. However, Allman et al. [13] demonstrated that ferrets, deafened as mature adults, exhibited a robust somatosensory reorganization of core auditory cortex, which included both the primary and anterior auditory fields. This single-unit recording study showed that neurons in the reorganized core auditory cortices were cutaneously driven, exhibited receptive fields located bilaterally on the head or head and neck, and lacked a global somatotopy. Thus, the core auditory cortex, described by so many studies as lacking visual crossmodal inputs (see above), is crossmodally innervated by somatosensory inputs following adult hearing loss. In fact, somatosensory reorganization of core auditory cortex was observed not only in response to profound deafening in adults [13], but in adult animals with incomplete hearing loss as well [14, 15]. However, because these mature animals were deafened long after their critical period for sensory development and plasticity (which ends near postnatal day 60 [16]), it could not be predicted whether the same crossmodal effects on core auditory cortex would also occur after early hearing loss. Therefore, the present experiments were designed to use the same experimental approaches as in the examination of the effects of adult-deafness, except that hearing deficits were induced early in the developmental sequence.

## 2. Materials and Methods

 All procedures were performed in compliance with the Guide for Care and Use of Laboratory Animals (National Institutes of Health, publication 86-23), the National Research Council's Guidelines for Care and Use of Mammals in Neuroscience and Behavioral Research (2003), and approved by the Institutional Animal Care and Use Committee at Virginia Commonwealth University. These procedures are the same as those used by Allman et al. [13] for examining the crossmodal properties of auditory cortical neurons in adult-deafened ferrets.

### 2.1. Deafening

**Figure 1:**
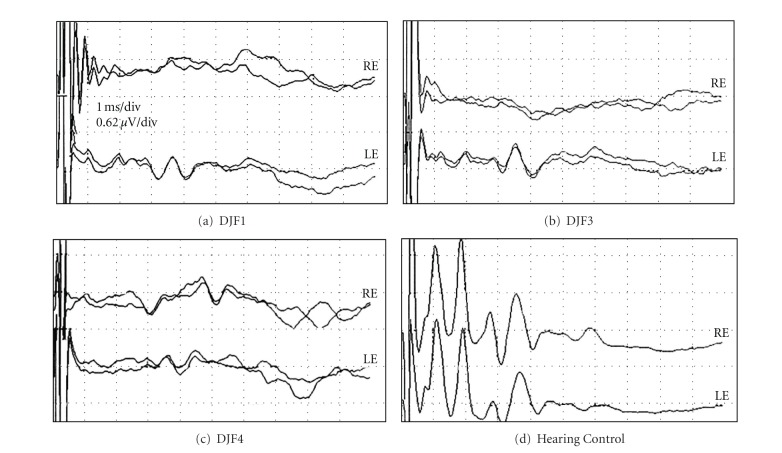
Auditory brainstem response (ABR) data for ferrets with early hearing loss (a–c) or normal hearing (d). For each panel, the auditory stimulus was a calibrated click (90 dB SPL; 0.1 ms square-wave click, rarefaction), delivered through a speaker positioned directly in front of one ear (RE = right; LE = left). Each waveform represents the average of 2000 trials; overlapped dual waveforms indicate that the test was repeated. Scale, indicated in panel (a), is the same for each panel. As evidenced by comparison of panels “a–c” with that of the hearing control in (d), all ferrets with early hearing loss demonstrated profoundly reduced ABRs to 90 dB SPL stimuli. However, some residual auditory response was apparent in each of the treated animals, as demonstrated by the small but repeatable peaks at approximately 3 ms latency. Further tests (not depicted) indicated that hearing thresholds for the treated animals ranged from 65 to 85 dB SPL (see Table 1).

 Ferrets begin to hear by the end of the first postnatal month [17], and primary auditory cortex reaches maturity one month later at approximately 60 days postnatally [16]. Therefore, to damage the functioning auditory system [18] before it matures, kanamycin (300 mg/kg, s.c.) and ethacrynic acid (25 mg/kg, i.v.; after protocol of [19]) were coadministered to ferrets (*n* = 3) on postnatal day 49 (see Table 1 for vital statistics). At approximately four weeks after ototoxic treatment, auditory brainstem responses (ABRs) were assessed for each ear separately, as shown in Figure 1. The auditory stimulus was a calibrated click (2000 trials each, 0.1 ms square-wave click, rarefaction), delivered through a speaker positioned in front of one ear. Subdermal recording leads were inserted over the right and left mastoid processes, at midcranium and on midback. Evoked electrical activity was signal averaged, and threshold response levels were determined using a descending (5–10 dB SPL increments) sequence of sound intensity for each ear of each animal. Bilateral ABRs were also tested on hearing animals (threshold ~15 dB SPL; reported in [13]).

### 2.2. Electrophysiology

At three to five months after the ototoxic procedure (see Table 1), the animals were at or near sexual maturity (150–180 days of age) and well beyond the critical period of auditory cortical development [16]. The early hearing-impaired ferrets were surgically prepared for electrophysiological recording. Under pentobarbital anesthesia (40 mg/kg, i.p.) and aseptic surgical conditions, a craniotomy was made over the left cortical hemisphere to expose the auditory cortices. Next, a stainless-steel well/head-support device was implanted using screws and dental acrylic, and the incision was closed around the implant. A standard postoperative antibiotic and analgesic regimen was administered, and the recording experiment occurred 2–4 days after implantation. Procedures and data from four, age-matched normal hearing ferrets (mean = 199 ± 4  DPN) also provided controls for another published study and are fully described there [13].

Electrophysiological recordings were initiated by anesthetizing the animal (35 mg/kg Ketamine; 2 mg/kg Acepromazine i.m.) and fixing the implanted well to a supporting bar. The animal was intubated through the mouth and ventilated with expired CO_2_ monitored and maintained at ~4.5%. Fluids, supplemental anesthetics (8 mg/kg/h Ketamine; 0.5 mg/kg/h Acepromazine), and a muscle relaxant (Pancuronium bromide 0.2 mg/kg/h i.p.) were continuously infused. This drug regimen was necessary to prevent spontaneous eye and body movements during the lengthy sensory/multisensory tests. The implant was opened, and the recording electrode (glass-insulated tungsten; <1 MΩ impedance at 1000 Hz) was inserted into core auditory cortex guided by gyral/sulcal landmarks and the functional map published by [20].

 With the electrode inserted into auditory cortex, neuronal activity was amplified and routed to a computer. Neurons were identified by their spontaneous activity and their responses to an extensive battery of manuallypresented auditory (claps, clicks, whistles, and hisses), visual (flashed or moving dark or light stimuli) somatosensory (strokes and taps using brushes and calibrated Semmes-Weinstein filaments; air puffs), manual pressure, and joint rotation) stimuli. Thus, at each location, the sensory response modality of the neuron (auditory, visual, somatosensory, multisensory, and unresponsive) was identified and tabulated, and the sensory receptive field(s) were mapped and graphically recorded. To reduce sampling bias during single-unit recording penetrations, neurons were studied at 250 *μ*m intervals. Due to their significant hearing loss, standard measures of auditory frequency-response tuning were not attempted in these experiments.

 Additional, quantitative sensory/multisensory tests were performed at selected recording sites. Quantitative sensory tests consisted of computer-triggered auditory, visual, and somatosensory stimuli, presented alone and in combination. Free-field auditory cues were electronicallygenerated white-noise bursts (100 ms, >80 dB SPL) from a hoop-mounted speaker 44 cm from the head (45° azimuth/0° elevation). Visual cues were bars of light projected onto a translucent hemisphere (92 cm diameter) whose size, direction, velocity, and amplitude were independently controlled. Somatosensory stimulation was achieved using an electronically-driven, modified shaker with independently programmable amplitude, and velocity settings to indent skin/deflect hairs. When no receptive field could be identified, a standard stimulus configuration was presented: auditory stimulus as described above; a large light bar (*5 × 15*°) that moved across the contralateral visual field from nasal to temporal at 200°/s); the somatosensory probe was positioned to stimulate the contralateral cheek. Each stimulus presentation was separated by 3–7 s, and each condition was presented 25 times. Neuronal responses were digitized (rate > 25 kHz), and individual waveforms were templated and routed to a computer for analysis. For each waveform (i.e., single neuron), a peristimulus-time histogram was constructed for each of the test conditions from which the response (mean spikes per trial) was measured. Unisensory neurons (auditory, visual, and somatosensory) were identified as those which were activated or influenced by only one sensory modality. Multisensory neurons activated by two different sensory modalities were defined as bimodal (e.g., auditory-somatosensory); those activated by three were classified as trimodal neurons. Multisensory neurons activated by one modality but whose response could be modulated (suppressed or facilitated) by a second modality that was ineffective alone were categorized as subthreshold neurons [15].

 The depth of each neuron within a penetration was noted and, in a data table, correlated with its sensory response type: unisensory auditory, visual, somatosensory, and multisensory combinations thereof, or unresponsive. Several recording penetrations were made in each animal, and their location was plotted on a digital photograph of the cortical surface. Each recording penetration was marked at its terminus with a small electrolytic lesion to facilitate its histological reconstruction. At the conclusion of the recording experiment, the animal received a barbiturate overdose (pentobarbital, 120 mg/kg, i.v.) and was perfused transcardially with saline followed by fixative (4% paraformaldehyde). The brain was removed from the cranium, and the auditory cortical regions were stereotaxically blocked and serially sectioned (50 *μ*m thick). The sections were processed using standard histological procedures, and a projecting microscope was used to make scaled reconstructions of the recording penetrations. Auditory cortical fields were approximated using sulcal landmarks according to the criteria of [20]. However, the sulcal borders of these fields have not been mapped. Furthermore, the adjoining sulci are known to contain non-auditory representations, such as the anterior and posterior lateral suprasylvian visual regions [21] or the pseudosylvian somatosensory region [22]. Therefore, a conservative approach was adopted to exclude data from these potentially non-auditory regions, whereby the core auditory cortices were defined by a border at the lip of the relevant sulcus.

### 2.3. Neuroanatomy

The cortical connectivity of the three early ototoxically-treated ferrets (the right hemisphere from the recorded animals) and four hearing adult ferrets was examined. Approximately 1 week prior to the terminal recording session and using pentobarbital anesthesia (40 mg/kg i.p.) and aseptic surgical conditions, a craniotomy was made to expose the right auditory cortices. Biotinylated dextran amine (BDA, 3k MW, 10% in citrate buffer) was pressure injected (0.8–1.5 *μ*L volume) from a 5 *μ*L Hamilton syringe into the A1 region using gyral/sulcal landmarks and the criteria of [20]. After a period for tracer transport, the animal was euthanized, perfused (saline) and fixed (4% paraformaldehyde). The brain was removed, blocked stereotaxically, and sectioned (50 *μ*m thick) in the coronal plane. Sections were processed using the protocol of [23] with metal intensification, mounted on slides, and coverslipped without counterstain. The processed sections were digitized using Neurolucida software (MBF Biosciences, Williston, VT, USA) to document the tissue outline, grey-white border, and the location of retrogradely labeled neuronal cell bodies. Functional maps of ferret cortex [20, 21, 24–26] and thalamus [27] were used to identify the regions containing retrogradely labeled neurons. For display, the sections were graphically arranged, and neural regions were identified using accepted sulcal/gyral/cytoarchitectonic landmarks [20, 26–28].

## 3. Results

Young ferrets (*n* = 3), ototoxically treated after hearing onset but well before the end of their critical period of auditory development, showed significant levels of hearing impairment as adults, as illustrated in Figure 1. Each treated animal showed an elevated hearing threshold of 65–85 dB SPL, which is summarized in Table 1. In contrast, the untreated hearing controls exhibited hearing thresholds of approximately 15 dB SPL [13].

**Figure 2:**
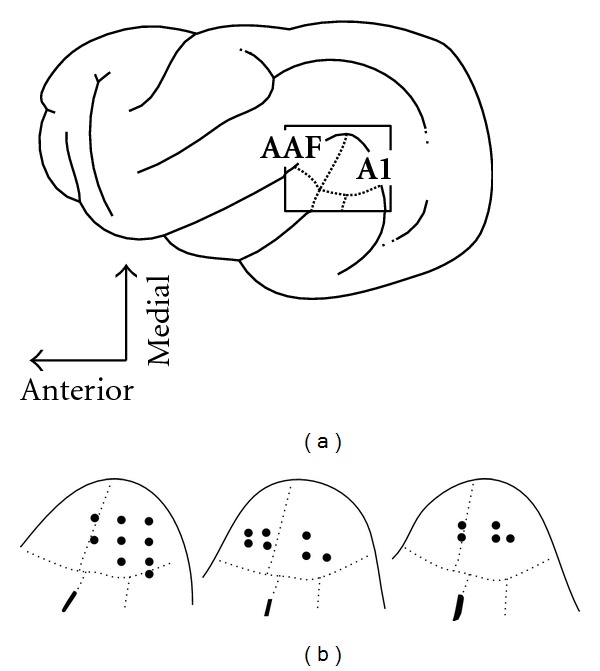
Single-unit recording penetrations in core (AAF and A1) auditory cortex of the three ferrets with early hearing loss. Part (a) shows a lateral view of the ferret cortex with the location of the core auditory areas of anterior auditory field (AAF) and primary auditory cortex (A1) indicated (boxed). Part (b) shows tracings of surface photographs of the positions of the recording penetrations (*n* = 21) made in each of the hearing impaired animals. Dotted lines approximate the borders of the auditory fields (after [20]).

To evaluate the sensory responsiveness of auditory cortical neurons in the hearing-impaired animals, single-unit recordings were made at sites across the upper/medial aspects of the middle ectosylvian gyrus (MEG), as depicted in Figure 2. This figure summarizes the cortical location of recording penetrations plotted from surface photographs for each animal. A total of 21 effective recording penetrations were made into the AAF or A1 regions. Because the animals were significantly hearing-impaired, however, the functional border between AAF and A1 could not be mapped, and the auditory cytoarchitectonic borders have not yet been described in this species. Hence, recordings were regarded as samples of the core (inclusive of AAF and A1) auditory cortices based on gyral/sulcal landmarks, histological reconstruction (see below), and published functional maps [20].

**Figure 3:**
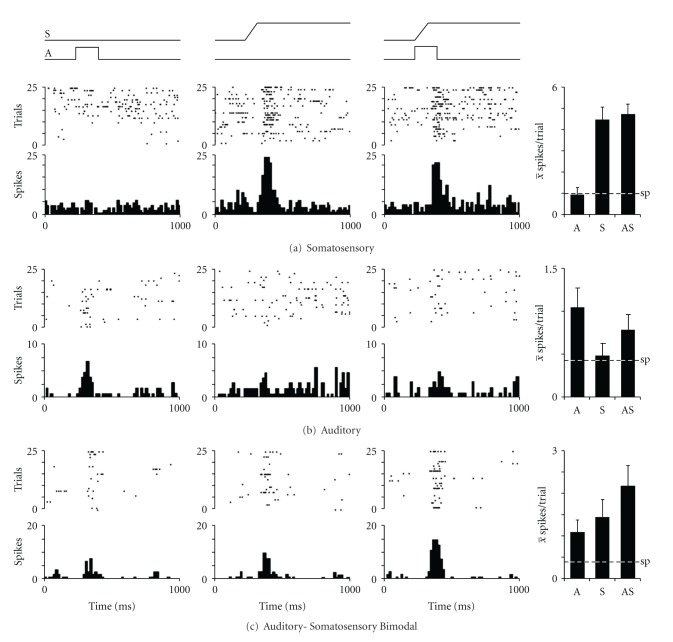
Sensory responses of neurons from core auditory cortex of ferrets with early hearing impairment. Single-unit recordings revealed that neurons were responsive to auditory (square wave, white noise), somatosensory (ramp; 1 g filament displacement of skin/hair) or combined auditory-somatosensory stimulation as displayed in the raster/histogram rows. In (a), the neuron was unresponsive to the auditory stimulus, but was vigorously activated by the somatosensory cue, and this response was not significantly altered when the two stimuli were combined. These responses are summarized by the bar graph (far right; error = standard error; sp = spontaneous activity), and are characteristic of a unisensory somatosensory neuron. The same conventions are used in the subsequent rows where activity indicative of a neuron with unisensory auditory properties (b) and a neuron with bimodal auditory-somatosensory response features (c) are illustrated. None of the depicted responses to combined stimulation were significantly (paired *t*-test) different from their best unisensory responses. These data show that core auditory cortical neurons in these hearing-impaired animals exhibit vigorous somatosensory-evoked activity.

Recording penetrations in the core auditory cortices of the early hearing-impaired ferrets revealed neurons that were responsive to somatosensory and/or auditory stimulation. As evidenced in the raster/histogram of Figure 3(a), some neurons were unresponsive to acoustic stimulation, but were robustly and reliably activated by a tactile stimulus presented within its receptive field. Furthermore, the tactile response was not significantly influenced when somatosensory and auditory stimulation was combined. Other neurons, like that represented in Figure 3(b), were responsive to acoustic stimulation, but were not significantly affected by tactile cues. In addition, many of the identified neurons were multisensory because they were activated by auditory stimulation alone as well as by independent somatosensory stimulation. An example of such a bimodal neuron is shown in Figure 3(c). All neurons were also tested for responsiveness to visual stimulation (see below); some neurons were encountered that were unresponsive to all the different stimuli and their combinations (not illustrated).

**Figure 4:**
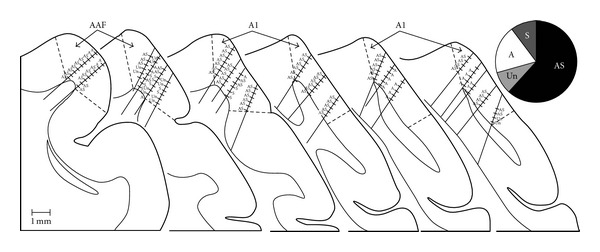
Coronal sections through core auditory cortex (AAF and A1) summarize each of the single-unit recording penetrations (*n* = 21) in ferrets with early hearing loss. For each penetration, the hashmarks indicate the location of an identified neuron (*n* = 132) whose functional properties are indicated: A = auditory; S = somatosensory; AS = auditory-somatosensory; Un = unresponsive. The pie chart (top right) summarizes the proportions of encountered neuron types: AS = 62%; S = 10%; A = 19%; and Un = 9%. The coronal sections are serially arranged (anterior : left) with the thin contour representing the gray-white border and the dashed lines indicating the presumed borders of AAF and A1. Because the sulcal borders of these regions have not been mapped, it was assumed that the sulcal extent of each area terminated at the lip of the sulcus. Only those neurons whose location plotted within the depicted borders of AAF/A1 were included in this study. Not all neurons are plotted due to overlap.

Neurons responsive to auditory as well as somatosensory cues were encountered across the MEG, as well as through the full thickness of the cortical mantle. Histological reconstructions of the recording sites (summarized in Figure 4) depict the location of each recording penetration and the neuron types that were encountered. Even though the recording penetrations clearly continued into white matter or the adjoining lateral bank of the suprasylvian sulcus, only those recording sites within the gyral aspects of the MEG were included for this study (see Section 2). When all neurons identified in 21 penetrations were tallied (*n* = 132), the overwhelming majority showed bimodal auditory-somatosensory properties (were independently activated by separate auditory and somatosensory stimulation; *62 ± 19*% sem; see pie chart in Figure 4). In addition, another 10% (±8 sem) were activated by somatosensory stimulation alone (e.g., unisensory somatosensory). Combining these values indicates that nearly 3/4ths of core auditory cortex in early hearing-impaired ferrets was responsive to somatosensory stimulation. In contrast, only 1 (1%; 1/100) somatosensory neuron was identified in similar recordings from normal hearing controls (from [13]).

In each case, every neuron was tested not only for auditory and somatosensory activation, but for visual inputs as well. In 6 of the penetrations, a total of 19 neurons responsive to visual stimulation were also identified. However, upon reconstruction of the recording tracks, these neurons were uniformly located at the deepest points of the penetration and within the bank of the suprasylvian sulcus that corresponds with the proposed lateral suprasylvian visual area of normal animals [21]. These visually-responsive neurons did not meet the criterion for residing within the gyral portion of core auditory cortex [20] and, therefore, were excluded from further analysis.

**Figure 5:**
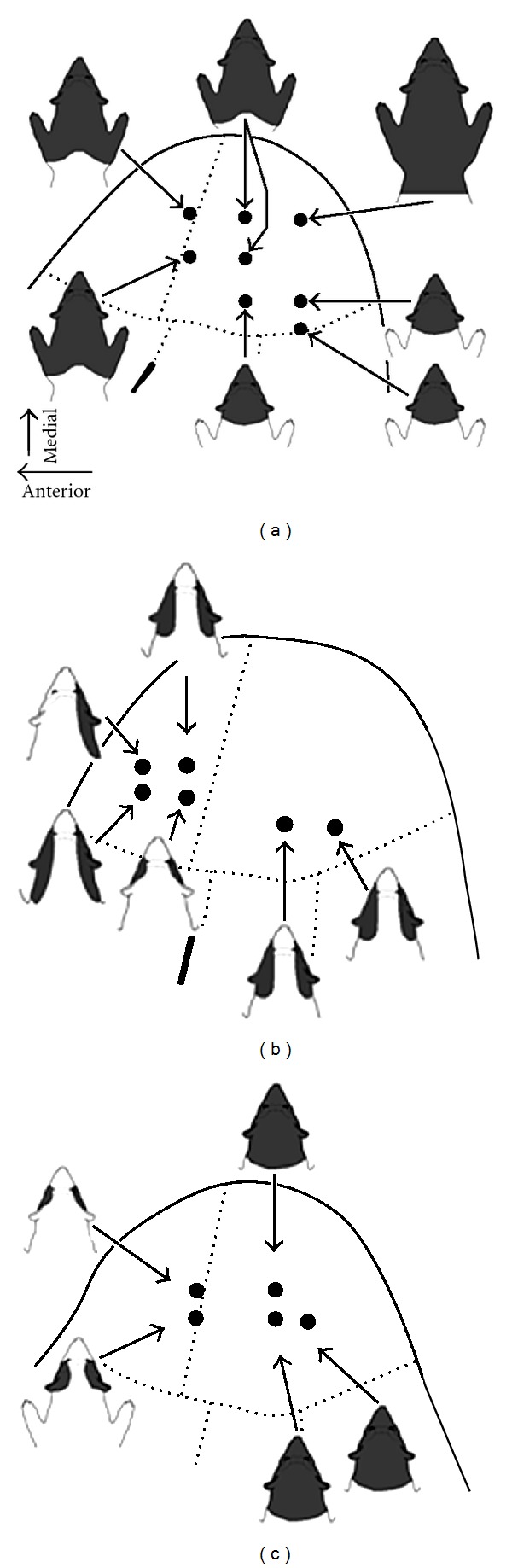
Distribution of somatosensory receptive fields in core auditory cortex of early hearing-impaired ferrets. For each of the surface plots for the different hearing-impaired animals (a–c), the associated schematic of the ferret's body surface shows the somatosensory receptive field(s) (shaded dark gray) encountered in that recording penetration. Note that in each case somatosensory receptive fields were located on the anterior aspect of the body (head, neck, and forelimb) and that they were predominantly bilateral (included both ipsi- and contralateral body surface).

**Figure 6:**
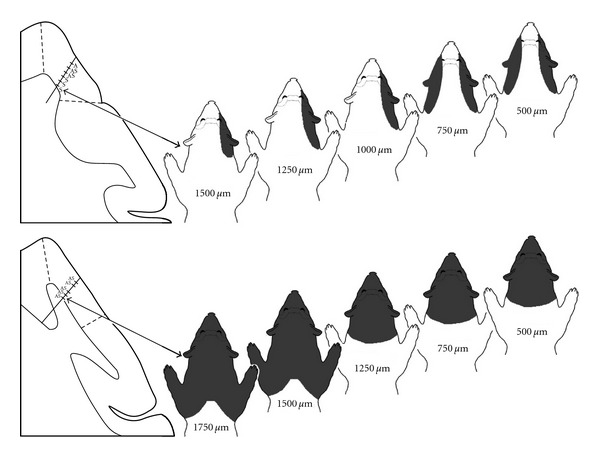
Distribution of somatosensory receptive fields across the cortical mantle (gray matter) of core auditory cortex in early hearing-impaired ferrets. Segments of coronal sections through core auditory cortex (left) show the location of a particular recording penetration and the sensory responsiveness of the neurons identified (A = auditory, S = somatosensory; AS = auditory and somatosensory). To the right, the series of ferret body surface depictions indicate the location of the somatosensory receptive field (shaded dark gray) and depth (in microns) corresponding to the neuron and recording penetration plotted on the tissue section. These data show that somatosensory reorganization of core auditory cortex in early hearing impaired ferrets was robust across the fullthickness of the cortex, represented the anterior segment of the body, and was often bilateral (on the ipsi- and contralateral body surface).

Somatosensory responses were recorded in the overwhelming majority (>80%; 17/21) of recording penetrations in the early hearing-impaired cortices, as shown in Figure 4. Somatosensory responses were observed through the full thickness of the cortical mantle, and representative somatosensory receptive fields observed for a given track are plotted in Figure 5. These data show for each animal that somatosensory receptive fields always included the face and often extended into other adjoining regions of the neck and/or forelimb and represented inputs carried by trigeminal and cervical nerves. In addition, the somatosensory receptive fields often represented bilateral aspects of the body surface including the standard contralateral representation as well as ipsilateral features. As also shown in the bilateral receptive fields depicted in Figure 5, their contralateral and ipsilateral distributions were usually symmetrical. Thus, as depicted in Figure 5(a), receptive fields that included the contralateral forepaw also included the same ipsilateral region, or in Figure 5(c) where the contralateral and ipsilateral pinnae are represented together in the same neuron. In addition, within a given recording penetration, somatosensory receptive fields tended to cluster around representation of a particular segment of the body surface. Two examples of this effect are provided in Figure 6. The recording penetration illustrated in Figure 6(a) occurs orthogonal to the pial surface and shows a recording sequence in which somatosensory receptive fields on the cheek, pinna, and neck were consistent along the depth of the recording penetration. However, the receptive fields shifted from bilateral to contralateral for the neurons located the deepest in the penetration. A similar vertically-oriented penetration depicted in Figure 6(b) also shows that somatosensory receptive fields were nested on the representation of the face, head, and neck, but abruptly expanded onto the bilateral forelimbs at the deepest recording sites.

**Figure 7:**
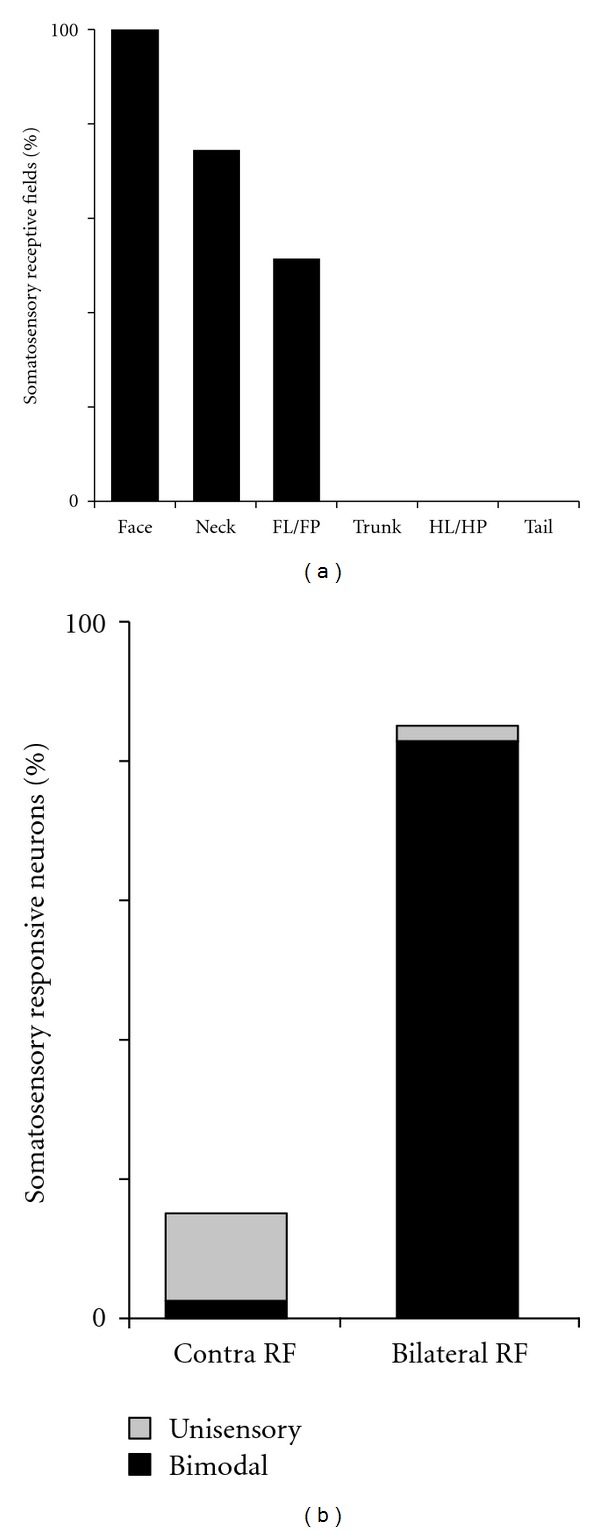
Somatosensory receptive field properties of neurons in core auditory cortex of early hearing-impaired ferrets. Part (a) shows that all somatosensory receptive fields included representation of the face and that these often extended into the neck or forelimb/forepaw (FL/FP) regions. Conversely, no somatosensory receptive fields were observed representing the posterior portion of the body (trunk, hindlimb/hindpaw (HL/HP), tail). Part (b) illustrates that the overwhelming majority of somatosensory receptive fields were bilateral (included ipsi- and contralateral body surface) of which 97% occurred in bimodal auditory-somatosensory neurons (black region of bar). In contrast, comparatively few neurons exhibited exclusively contralateral somatosensory receptive fields, most of which (86%; grey region of bar) occurred in unisensory somatosensory neurons.

When all of the mapped somatosensory receptive fields were analyzed, it became apparent that all somatosensory-responsive neurons had receptive fields that included the face, as is summarized in Figure 7(a). It was also revealed that many somatosensory receptive fields could also include the neck, or neck and forelimb/forepaw. However, no receptive fields that included the trunk, hindlimb/paw or tail were encountered. Also a laterality component of receptive field distribution that strongly correlated with the unisensory/multisensory nature of the parent neuron was observed. As shown in Figure 7(b), bilateral receptive fields predominated over those with purely contralateral representations (85 : 15 ratio). Furthermore, 86% of unisensory somatosensory neurons exhibited receptive fields with contralateral distributions, while 97.5% of bimodal neurons demonstrated bilateral receptive fields. Most (65%) somatosensory neurons were excited by very low force threshold stimulation of ≤1* *gram, which is consistent with activation of peripheral hair receptors.

**Figure 8:**
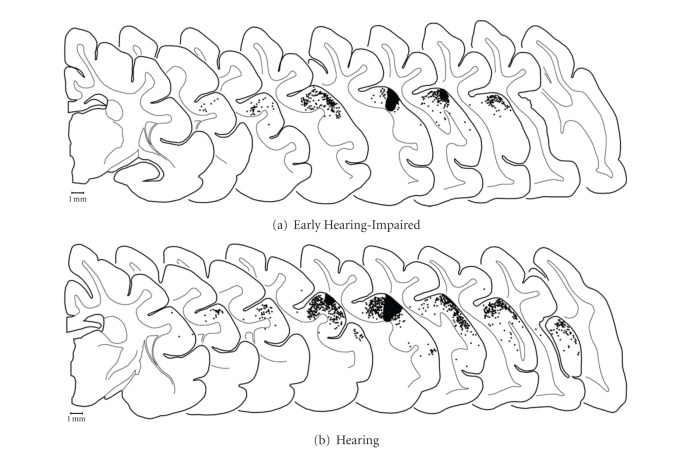
Sources of cortical inputs to core auditory cortices for early hearing-impaired (a) and normal hearing (b) ferrets. On serially arranged (anterior = left) coronal sections through one cortical hemisphere, tracer injection (BDA; solid black area) into core auditory cortex produced retrogradely labeled neurons (1 dot = 1 neuron) primarily within the regions regarded as auditory cortex: on the middle ectosylvian gyrus (MEG) and the posterior aspect of the anterior ectosylvian gyrus (AEG), as well as within the lateral bank of the suprasylvian sulcus (SSS) and into the bank of the pseudosylvian sulcus (PSS). The distribution of labeled neurons is essentially coextensive for hearing-impaired and hearing animals, with no difference in labeling found within somatosensory regions on the suprasylvian (SSG) and anterior ectosylvian gyri. Hearing controls replotted from [13].

These recordings in early hearing-impaired ferrets indicate that approximately 72% of core auditory cortical neurons are responsive to somatosensory stimulation. In contrast, using the same recording methods, only 1% of neurons from hearing controls showed the same somatosensory sensitivity [13]. Therefore, it seemed possible that during and following postnatal development, a large contingent of novel somatosensory inputs reached the auditory cortices of the early hearing-impaired animals. To examine whether these crossmodal inputs arrive from somatosensory cortical sources, the core auditory cortex of these same early hearing-impaired ferrets (*n* = 3) received tracer (BDA) injections, and the loci of the resulting retrogradely labeled neurons were plotted. A representative example is presented in Figure 8(a), which shows that sources of inputs to early hearing-impaired arose largely from other auditory cortices, and the somatosensory and visual regions were essentially devoid of label. This pattern of labeling was consistent for 2 of the 3 cases. In the third case, the injection was more extensive and included not only the subadjacent white matter, but also aspects of the medial bank of the pseudosylvian sulcus known to receive visual inputs [22] and contains the anterior ectosylvian visual area [26]. In this case, the same auditory cortical areas revealed retrograde labeling, but visual areas 19, 20a, and 20b (after [28]) were also labeled. However, and none of the three cases were more than a few labeled neurons found in any of the somatosensory cortical areas. Collectively, these data demonstrated that cortical projections to core auditory cortex of early hearing-impaired ferrets were almost entirely from other auditory cortical areas, just like they were for normal hearing ferrets (see Figure 8(b) of [13]).

**Figure 9:**
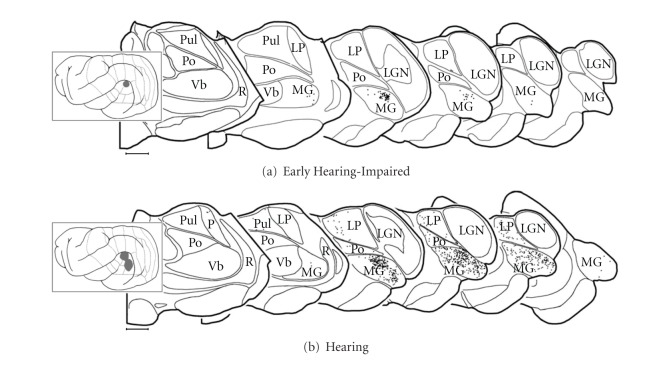
Sources of thalamic inputs to core auditory cortices for early hearing-impaired (a) and normal hearing (b) ferrets. On serially arranged (anterior:left) coronal sections through the thalamus of an early hearing-impaired ferret, tracer injection (BDA; solid grey area) into core auditory cortex produced retrogradely labeled neurons (1 dot = 1 neuron) essentially within the regions regarded as auditory thalamus: in the medial geniculate nucleus (MG). No retrgradely labeled neurons were identified in somatosensory (Vb) or visual (LGN) thalamus although a few were found in the border of the lateral posterior (LP) and posterior (Po) nuclei. Although a larger injection in a hearing animal encroached on the secondary auditory fields, the thalamic labeling was focused on the MG, with lesser connectivity with the LP and Po; no labeled neurons were observed in somatosensory (Vb) or visual (LGN) thalamic nuclei. Hearing controls replotted from [13].

To assess whether crossmodal inputs to auditory cortex of early hearing-impaired ferrets might arise from somatosensory thalamus, the same cases described above were used to plot the location of labeled thalamocortical neurons. As illustrated in Figure 9(a), a small injection into core auditory cortex of an early hearing-impaired animal almost exclusively yielded retrogradely labeled neurons within the medial geniculate nucleus of the auditory thalamus. This pattern of labeling was consistent for 2 of the 3 cases. In the third case, the injection was more extensive and extended into the visual area of the pseudosylvian sulcus (AEV; [22, 26]). In this case, labeled thalamocortical neurons were more frequent within the LP and Po regions, and a few were observed in the visual LGN or in the posterior-lateral aspects of the somatosensory VB. In all early hearing-impaired cases, however, the overwhelming preponderance of thalamic projections to core auditory cortex was from the medial geniculate, which was consistent with that observed in normal hearing ferrets (Figure 9(b) of [13]).

## 4. Discussion

These data show that early hearing loss results in crossmodal reorganization of core auditory cortex, such that neurons normally driven by auditory inputs respond to somatosensory stimulation. The novelty and importance of this observation resides in the context of the literature of crossmodal plasticity following sensory loss. First, many studies have reported a lack of crossmodal innervation of core auditory cortex in early-deaf subjects [2, 4–7, 12]. However, these studies tested for visual responses and, hence, established only that visual crossmodal effects were not observed. In contrast, when early-deaf subjects were examined using somatosensory stimulation, crossmodal plasticity of the core auditory areas was observed in humans [29] and animals (present study). In addition, the present results from early hearing loss closely correspond with that observed in animals with late, or adult, hearing loss [13–15]. In both conditions (early- and late-hearing loss), the preponderance of single-unit recordings from neurons in core auditory cortex was demonstrated to exhibit robust responses to somatosensory inputs. Therefore, rather than being immune to the plasticity that occurs elsewhere in the auditory cortices following hearing loss, core auditory cortex actually exhibits crossmodal effects in the form of somatosensory reorganization.

Single-unit studies of crossmodal plasticity provide unique insights into the features of the reorganizing modality. Not only do neurons in core auditory cortex of early hearing-impaired animals respond to somatosensory stimulation, the majority of them are activated by low force-threshold receptors corresponding to hair-type receptors. In addition, the receptive fields of the individual, crossmodally innervated neurons, could be mapped. These data showed that all such receptive fields included the head/face, and many also extended onto the neck and/or forelimb. Thus, there was a strong preference for representation of the anterior aspects of the body surface conveyed by trigeminal/cervical nerves, and no receptive fields were encountered that included the hindlimb or tail. This information suggests that the fMRI study of congenitally deaf humans may have activated even larger proportions of core auditory cortex had the test stimuli been applied to the face rather than the hands [29]. Furthermore, over 80% of the receptive fields were bilateral in distribution, such that they included both contralateral and ipsilateral portions of the body surface. The bilateral arrangement of these receptive fields, and their preponderance in the present sample, are quite unlike the contralateral distribution of receptive fields encountered in somatosensory cortical areas of ferret SI–SIII [24, 25, 30] and the medial rostral suprasylvian somatosensory area [31]. Collectively, it is difficult to discern a pattern of somatotopy from these results, given that the same receptive field locations are represented at widely different locations within core auditory cortex (e.g., see Figure 5). Ultimately, these somatosensory features of reorganized auditory cortex in early hearing-impaired ferrets are fundamentally similar to those observed in late-, or adult-onset deaf [13] or hearing-impaired ferrets [14, 15]. Consistent among each of these types of hearing loss, core auditory cortical crossmodal plasticity was characterized by somatosensory reorganization that was activated largely by low force-threshold hair type receptors and represented the head/anterior body surface, often bilaterally, without apparent global somatotopy. At present, it is difficult to postulate how such reorganization might convey “adaptive” or “compensatory” advantages at neuronal or perceptual levels, but instead may contribute to the growing literature that interprets some forms of crossmodal plasticity as “maladaptive,” such as tinnitus (reviewed in [32]).

Given the overwhelming presence of somatosensory-responsive neurons in core auditory cortex of these early hearing-impaired animals, it would be expected that robust connections with somatosensory brain regions would be established in these altered animals. In addition, because a large proportion of crossmodal studies have identified changes specifically in cortical function (reviewed in [2, 33]), novel cortical connections between core auditory cortex and portions of somatosensory cortex would seem most likely to occur. However, tracer injections placed in core auditory cortex of early hearing-impaired animals revealed few, if any, inputs from somatosensory cortex and did not appear to be different from core auditory cortical connections in hearing control animals. Furthermore, thalamocortical connections did not reorganize in the early hearing-impaired animals as the somatosensory thalamic nuclei were essentially devoid of label. These data indicate that changes in regional cortical and thalamocortical connectivity were not sufficient to underlie the wholesale functional changes observed in core auditory cortex of early hearing-impaired ferrets. Ultimately, these connectional data in early hearing-impaired ferrets are essentially identical to those observed for the somatosensory reorganized core auditory cortex of adult-deafened ferrets [13].

Because the neuroplastic effects that occur during the developmental critical period of young animals or subjects are well known, it might be expected that crossmodal effects in early lesioned subjects would differ from those whose onset occurs during adulthood. However, crossmodal plasticity has only rarely been examined in adults. Sound localization behavior in late visually-deprived cats was observed to be similar to, but not as strong as that demonstrated by early-deprived animals [34]. Likewise, sound localization in early blind [35] and late-blind individuals [36] was behaviorally similar, but appears to involve different components of the neural response [36]. The present study of crossmodal functional and connectional effects in early hearing-impaired ferrets closely resembles the results from adult-deaf [13] or adult hearing-impaired ferrets [14, 15]. Collectively, these studies represent, to the best of our knowledge, among the only single-unit or neuroanatomical studies of cortical crossmodal plasticity precipitated by hearing loss (see also [11]). Despite the different stages of maturity and different severity of hearing loss involved, the results were quite similar; the core auditory cortex exhibited a functional somatosensory reorganization that was not accompanied by regional changes in connectivity. As discussed below, only a new theory of cortical crossmodal plasticity can account for these combined observations.

The mechanism underlying crossmodal plasticity has been of considerable interest. More than a decade ago, Rauschecker [33] summarized the known possibilities as follows: “[Crossmodal] plasticity might involve any or all of these neural mechanisms: unmasking of silent inputs; stabilization of normally transient connections, axonal sprouting; or a combination of them.” However, recent data is difficult to reconcile with these earlier postulates. With regard to unmasking of silent inputs, such crossmodal inputs should be revealed by connectional studies of auditory cortex in normal hearing animals. In neither the ferret (present studies) nor the cat [37–39] is there sufficient connectivity from somatosensory structures to underlie the robust somatosensory reorganization of the entire auditory area although the cortex of rodents appears to have a higher proportion of natural crossmodal connections [40, 41]. Alternatively, if crossmodal plasticity was subserved by the preservation of transient connections, then core auditory connectivity should be different between the early hearing-impaired and the normal hearing animals. The present study demonstrated that they were not fundamentally different. Furthermore, this particular mechanism could not account for the somatosensory reorganization observed in adult-deafened ferrets [13]. Last, in the present experiments, there was no evidence of sprouting of axons (e.g., ingrowth of new connections) from somatosensory cortical or thalamic regions in either the early- or late hearing-impaired animals sufficient to underlie the observed crossmodal effects. Therefore, an alternate hypothesis for the mechanism underlying early and late-deafness-induced crossmodal plasticity has been proposed by Allman et al. [13]. This theory accounts for the functional reorganization of auditory cortex in the deaf without requiring the actual plasticity to occur within the cortex. As has been known for over a decade now, the auditory brainstem naturally receives crossmodal inputs from the somatosensory system at several critical nodes. Neurons in the dorsal cochlear nucleus [42–44] as well as the inferior colliculus [45] have been demonstrated to be affected by somatosensory stimulation or by activation of the trigeminal nucleus. Furthermore, a recent study [46] showed that hearing loss enhances the level of crossmodal somatosensory innervation of the dorsal cochlear nucleus, where significant decreases in response thresholds as well as latency and duration changes were evident. Thus, cochlear damage results in the loss of functional auditory inputs to the cochlear nucleus and induces somatosensory crossmodal plasticity there. Because the cochlear nucleus is the first node in the ascending auditory projection, any functional changes within that nucleus are reflected throughout the entire auditory pathway, including cortex. This postulate is consistent with the representation of trigeminal and cervical somatosensory regions in deafened auditory cortex. Furthermore, given the highly crossed nature of the ascending auditory projection, it is not surprising that a high proportion of crossmodal somatosensory receptive fields in auditory cortical neurons are bilateral. Thus, the brainstem theory of cortical crossmodal *reorganization* (not *plasticity*), while being a significant departure from earlier postulates, seems well supported by empirical observations from different published sources and points of view. Further studies are necessary to map the specific somatosensory or visual spheres of crossmodal influence and determine how they might be appropriated.

## 5. Conclusions

Sensory loss, such as deafness, is well known to induce crossmodal changes involving the remaining sensory modalities. Many such studies have documented the presence of visual activation of secondary, but not core, auditory cortices following early deafness. The present study demonstrates that core auditory cortices also exhibit crossmodal reorganization following early hearing loss, but through the somatosensory modality. Because similar core auditory cortical effects occur as a result of early or adult hearing loss that do not conform with assumed mechanisms for crossmodal plasticity, a new brainstem theory of cortical crossmodal reorganization is proposed.

## Figures and Tables

**Table 1:** Hearing and age statistics for the ferrets with early hearing loss induced by ototoxic lesion (OT); all animals were male. The age of hearing onset in ferrets is ~32 days postnatally, and auditory critical period closure for A1 is 80 days [16].

ID no.	Weight	Age at OT lesion	ABR hear threshold	Age at recording	Impairment duration	BDA volume
DJF 1	1.7 kg	49 days	65 dB SPL	148 days	99 days	0.5 *μ*L
DJF 3	2.3 kg	49 days	65 dB SPL	182 days	133 days	0.5 *μ*L
DJF 4	1.9 kg	49 days	85 dB SPL	190 days	141 days	0.5 *μ*L
